# Transmissibility of SARS-CoV-2 B.1.1.214 and Alpha Variants during 4 COVID-19 Waves, Kyoto, Japan, January 2020–June 2021

**DOI:** 10.3201/eid2808.220420

**Published:** 2022-08

**Authors:** Yasufumi Matsumura, Miki Nagao, Masaki Yamamoto, Yasuhiro Tsuchido, Taro Noguchi, Koh Shinohara, Satomi Yukawa, Hiromi Inoue, Takeshi Ikeda

**Affiliations:** Kyoto University Graduate School of Medicine, Kyoto, Japan (Y. Matsumura, M. Nagao, M. Yamamoto, Y. Tsuchido, T. Noguchi, K. Shinorara, S. Yukawa);; Health and Welfare Bureau of Kyoto City, Kyoto (H. Inoue, T. Ikeda)

**Keywords:** COVID-19, respiratory infections, severe acute respiratory syndrome coronavirus 2, SARS-CoV-2, SARS, coronavirus disease, zoonoses, viruses, coronavirus, contact tracing, household contact, epidemiology, viral genome, Japan

## Abstract

Household transmission is a primary source of SARS-CoV-2 spread. We used COVID-19 epidemiologic investigation data and viral genome analysis data collected in the city of Kyoto, Japan, during January 2020–June 2021 to evaluate the effects of different settings and viral strains on SARS-CoV-2 transmission. Epidemiologic investigations of 5,061 COVID-19 cases found that the most common category for close contact was within households (35.3%); this category also had the highest reverse transcription PCR positivity. The prevalent viral lineage shifted from B.1.1.214 in the third wave to the Alpha variant in the fourth wave. The proportion of secondary cases associated with households also increased from the third to fourth waves (27% vs. 29%). Among 564 contacts from 206 households, Alpha variant was significantly associated with household transmission (odds ratio 1.52, 95% CI 1.06–2.18) compared with B.1.1.214. Public health interventions targeting household contacts and specific variants could help control SARS-CoV-2 transmission.

Robust testing, isolation, and epidemiologic investigations of patients and their close contacts by local public health authorities are key strategies for containing SARS-CoV-2 transmission (*1*). In response to the COVID-19 pandemic, the Ministry of Health, Labour and Welfare in Japan, according to law, implemented an all-case tracing approach that included mandatory reporting of laboratory-confirmed COVID-19 cases, case investigations, and contact tracing. In Japan, after outbreaks on cruise ships and identification of imported cases, COVID-19 clusters were reported in healthcare and long-term care facilities (LTCFs), restaurants, workplaces, and events, and those became the main target of COVID-19 interventions (*2*,*3*). Households have become the main venue for community transmission (*4*,*5*), and household contacts have a higher risk for secondary infection than nonhousehold contacts (*6*). Moreover, household transmission could be increasingly relevant during periods of social distancing and stay-at-home orders (*7*).

Specific SARS-CoV-2 variants, namely, those designated variants of concern (VOCs), generally have higher transmissibility than non-VOCs. The Alpha VOC was estimated to have a reproduction number 43%–90% higher than previous variants and has spread worldwide, including throughout Japan (*8*).

Kyoto, an ancient capital city of Japan, has a population of ≈1 million and is known as a tourist destination. By June 2021, Kyoto had experienced 4 waves of COVID-19. In response to these waves, the Health and Welfare Bureau of Kyoto City and a tertiary referral hospital of Kyoto University Hospital, which has infectious disease and clinical laboratory specialists, collaborated to perform epidemiologic investigations, establish interventions for cluster-associated cases, and conduct molecular epidemiologic surveillance. We describe COVID-19 epidemiology in Kyoto and focus on the effects of cluster and household transmission of different SARS-CoV-2 variants.

## Materials and Methods

### Active Epidemiologic Investigations

The Health and Welfare Bureau of Kyoto performed active epidemiologic investigations of all laboratory-confirmed COVID-19 cases in the city according to the guidelines of the National Institute of Infectious Diseases, Japan (*9*). These investigations collected the clinical data of COVID-19 patients, behavioral histories for 14 days before symptom onset or diagnosis, and detailed activity histories for 2 days before symptom onset or diagnosis. On the basis of those data, the bureau conducted contact tracing by identifying potential sources of infection and close contacts. The epidemiologic study was determined to be public health surveillance as defined in Article 15 of the Act on the Prevention of Infectious Diseases and Medical Care for Patients with Infectious Diseases (1999); thus, informed consent was not required.

The bureau defined close contacts as persons who lived with a COVID-19 patient or who had been <2 m from a patient for >15 min without using necessary preventive measures, such as personal protective equipment, within 2 days before the COVID-19 patient’s symptom onset or diagnosis (*9*). All household members of COVID-19 patients were considered close contacts (*9*). The bureau requested that close contacts quarantine for at least 14 days and get 1 reverse transcription PCR (RT-PCR) test at the beginning of the quarantine regardless of symptoms. 

We defined a cluster as identification of >5 cases at the same facility or among a group of contacts within 14 days of symptom onset or diagnosis for any patient, excluding household contacts. For clusters and cases that occurred in high-risk settings such as healthcare facilities, non–close contacts who shared a space with COVID-19 case-patients, such as in a workplace, also underwent RT-PCR testing to identify asymptomatic cases. 

We obtained epidemiologic data from existing local databases, and we determined the RT-PCR test positivity of close contacts according to the source of infection. We compared the number of household transmissions and the number of clusters between the third and fourth COVID-19 waves in Kyoto and between SARS-CoV-2 variants. 

### Epidemiologic Data

We obtained data on the number of COVID-19 cases in Kyoto and in Japan during January 2020–June 2021 from official websites for Kyoto City and the Ministry of Health, Labour and Welfare, Japan (*10*,*11*). We also obtained data from these websites on the number of persons who received RT-PCR testing at the official laboratories of Kyoto City or Kyoto Prefecture and commercial laboratories.

### Clinical Samples

Respiratory tract samples that tested positive by RT-PCR were sent to the reference laboratory at Kyoto University and subjected to genome analysis. The samples were obtained from RT-PCR testing sites, acute-care hospitals, close contacts found by active epidemiologic investigations, and mass PCR testing for residents and workers of adult daycare and LTCFs.

### Genome Analysis

We prepared a genome library by using an amplicon-based next-generation sequencing assay, the research-use-only COVIDSeq Test (RUO Version; Illumina, https://www.illmina.com), and sequenced samples by using the NovaSeq6000, NextSeq1000, NextSeq550, or MiniSeq platforms (Illumina). We processed the data by using DRAGEN COVID Lineage App version 3.5.3 (Illumina), and generated consensus sequences by using the SARS-CoV-2 reference genome (GenBank accession no. NC_045512). Using Pangolin version 3.1.20 (*12*), we assigned lineages to sequenced genomes that had >90% breadth of coverage of the reference genome and for genome data from Japan obtained from the GISAID database (https://www.gisaid.org) on July 13, 2021. We defined VOCs according to the World Health Organization designations as of June 22, 2021 (*13*). We used IQ-TREE multicore version 2.1.2 COVID-edition (http://www.iqtree.org) for phylogenetic analysis. We submitted SARS-CoV-2 sequences obtained in this study to GISAID (Appendix 1).

### Statistical Analysis

We calculated the secondary attack rate (SAR) by dividing the number of secondary cases within 14 days of the index case-patients’ positive RT-PCR test date by the total number of household contacts. We defined the index case as the first laboratory-confirmed case in the household. We excluded households in which coprimary cases had the same symptom onset date or same diagnosis date as primary cases. To analyze the association between SARS-CoV-2 variant and household transmission and to predict SAR, we used a generalized linear mixed-effects logistic regression model. In this model, we used random intercepts to account for clustering by household, the dependent variable of SARS-CoV-2 infection of contacts, and the predictors of the age of the index case-patient, the age of the contact, the presence of symptoms in the index case-patient, the household size, and the SARS-CoV-2 lineage, as previously described (*14*).

We used Fisher exact test to compare the categorical variable sex and Mann-Whitney U test to compare the continuous variable age. We considered p<0.05 statistically significant. We conducted statistical analyses by using R version 4.1.3 (R Foundation for Statistical Computing, https://www.r-project.org). The Ethics Committee of Kyoto University Graduate School and the Faculty of Medicine approved this study (approval no. R2379) and waived the need to obtain informed consent from study subjects.

## Results

**Figure 1:**
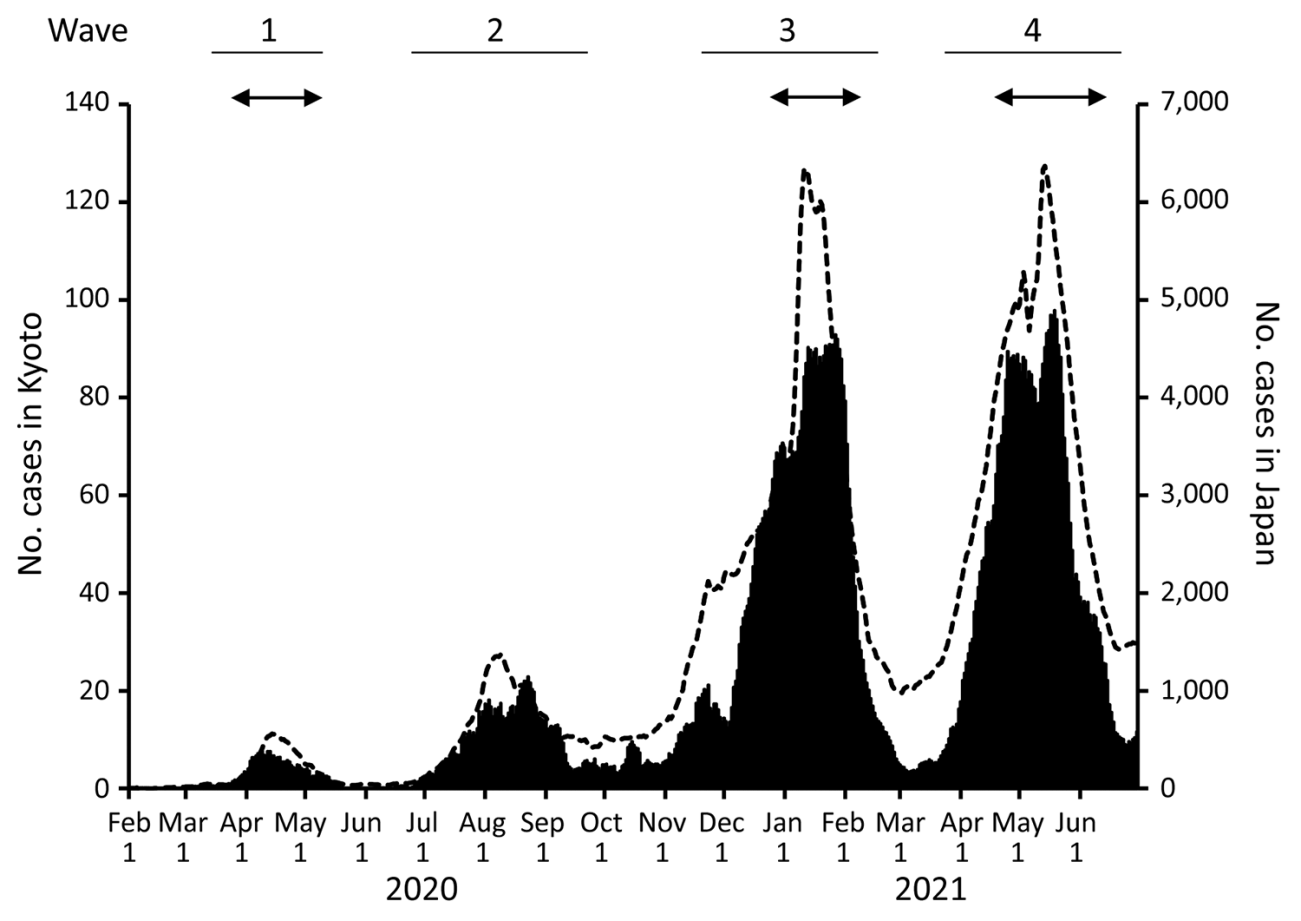
Seven-day moving average of cases during 4 COVID-19 waves, Kyoto, Japan, January 2020–June 2021. Solid black represents averages in Kyoto City and dashed lines represent averages in Japan. Arrows indicate the state of emergency designation in Kyoto Prefecture, in which Kyoto is located. Scales for the y-axes differ substantially to underscore patterns but do not permit direct comparisons.

During January 2020–June 2021, Japan had a total of 792,256 reported COVID-19 cases, among which 11,477 cases were reported in Kyoto (Figure 1). Japan and the city of Kyoto experienced 4 COVID-19 waves during that period. The third (December 2020–February 2021) and fourth (April 2021–June 2021) waves were larger than the first (April 2020–May 2020) and second (July 2020–September 2020) waves (Figure 1). 

**Figure 2:**
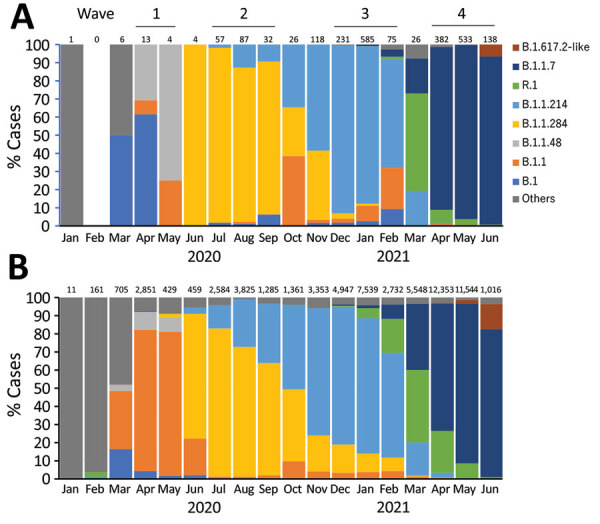
Prevalence of major SARS-CoV-2 viral lineages detected among respiratory tract specimens collected during 4 COVID-19 waves in Japan, January 2020–June 2021. A) Lineages detected in Kyoto City. B) Lineages detected from 62,703 genomes obtained in Japan and downloaded from the GISAID database (https://www.gisaid.org). Number of available genomes analyzed per month is shown above each bar. The most common lineages during each wave in Kyoto were B.1 (n = 8, 47.1%) during the first wave; B.1.1.284 (n = 156, 88.6%) during the second; B.1.1.214 (n = 766, 86.0%) during the third; and B.1.1.7 (Alpha; n = 983, 93.4%) during the fourth. B.1.48 was the second most common lineage during the first wave (n = 7, 41.2%) and R.1 was the most common lineage during March 2021 (n = 14, 53.8%), between the third and fourth waves. The most common lineages during each wave in Japan were B.1.1 (n = 2,561, 78.1%) during the first wave; B.1.1.284 (n = 5,641, 73.3%) during the second; B.1.1.214 (n = 10,970, 72.1%) during the third; and B.1.1.7 (Alpha; n = 19,630, 78.8%) during the fourth. B.1.48 was the second most common lineage during the first wave (n = 313, 9.5%) and R.1 was the second most common lineage during March 2021 (n = 2,217, 40.0%).

We performed genomic analysis on a total of 2,600 nonduplicate samples, representing 22.7% of COVID-19 cases in Kyoto. We determined pangolin lineages for 2,318 samples, the median coverage of which was 99.7% (interquartile range [IQR] 99.1%–99.8%) of the reference genome. The primary lineage responsible for each wave in Kyoto shifted during the 4 waves, from B.1 (47.1%) during the first wave to B.1.1.284 (88.6%) in the second, B.1.1.214 (85.4%) in the third, and B.1.1.7 (Alpha; 93.4%) in the fourth (Figure 2, panel A). During March 2021, between the third and fourth waves, R.1 was the most common (53.8%) lineage. We noted 2 VOCs: Alpha lineages B.1.1.7 and Q.1 (n = 998) during January 2021–June 2021 and Delta B.1.617.2-like (n = 9) during July 2021. The prevalent lineages in the 4 COVID-19 waves in Kyoto were the same as the rest of the country, except Japan had B.1.1 dominance during the first wave (Figure 2, panel B).

Active epidemiologic investigation of 5,061 COVID-19 cases in Kyoto identified 13,562 close contacts during November 2020**–**March 2021. The most common contact categories were household contacts (35.3%) and family living separately (10.4%), followed by workplace (staff 17.6% and user 8.2%), school (12.9%), and friend contacts (11.5%). Of close contacts, 11,813 (87.1%) had RT-PCR tests and 15.1% tested positive (Table 1). Test positivity was the highest among household contacts (24.9%) and friend contacts (16.4%). The test positivity rate among the general population during the same period was 5.9%.

**Table 1:** Reverse transcription PCR test positivity for close contacts of 5,061 COVID-19 case-patients, Kyoto, Japan, November 2020–March 2021

Contact category	No. identified	No. (%) tested	No. (%) positive
Household members living together	4,789	4,523 (94.4)	1,128 (24.9)
Family living separately	1,415	1,038 (73.4)	150 (14.5)
School, including nursery	1,755	1,696 (96.6)	38 (2.2)
Workplace, working staff	2,384	1,966 (82.5)	169 (8.6)
Workplace, user	1,114	1,030 (92.5)	67 (6.5)
Friend	1,566	1,174 (75.0)	193 (16.4)
Others	539	386 (71.6)	39 (10.1)
Total	13,562	11,813 (87.1)	1,784 (15.1)

We assessed the association of COVID-19 cases with household transmission events and clusters during the third and fourth waves in Kyoto (Table 2). Compared with cases in the third wave, the mean number of cases per day was higher during the fourth wave (51.0 vs. 68.5), as was the percentage of household cases among all cases (26.7% vs. 28.9%). The percentage of cluster-associated cases among all cases was higher during the third wave than during the fourth wave (15.4% vs. 10.7%), as was the median number of cases associated with each cluster (11 vs. 7), but the mean number of clusters per day was higher during the fourth wave than the third wave (0.70 vs. 0.44). The most common settings for clusters during the third wave were welfare facilities (60.0%) and hospitals (25.0%), but during the fourth wave, offices (30.2%) and adult daycare and LTCFs (16.3%) were the most common settings (Appendix 2 Table).

**Table 2:** COVID-19 cases associated with households or clusters during the third and fourth disease waves, Kyoto, Japan*

Variables	Third wave	Fourth wave
Date	2020 Dec–2021 Feb	2021 Apr–May
Total no. cases (mean no./d)	4,592 (51.0)	4,181 (68.5)
Total no. (%) secondary cases among households	1,228 (26.7)	1,208 (28.9)
Total no. (%) cases associated with clusters	849 (18.5)	449 (10.7)
Median no. cases associated with each cluster (IQR)	11 (9–20.5)	7 (6–9)
Total no. clusters (mean no./day)	50 (0.56)	43 (0.70)
Median test positivity of each cluster, % (IQR)†	18 (9.7–37)	34 (15.6–66.3)

*IQR, interquartile range. †Calculated by using epidemiologic data available for 48 clusters in the third wave and 35 clusters in the fourth wave.

Because Alpha was the main variant during the fourth wave, we compared the household transmission rates associated with the Alpha VOC and the non-VOC B.1.1.214. We investigated 310 households in which >1 member was infected with SARS-CoV-2 during November 2020–May 2021, regardless of symptoms, and for which all members had RT-PCR testing. We noted 245 households in which >1 household member was infected with Alpha or B.1.1.214 SARS-CoV-2 lineages. We excluded 29 households with incomplete demographic data, 9 households with >1 index case, and 1 household with a contact infected >14 days after the symptom onset or diagnosis of the index case. Thus, we found 206 households eligible for comparison, 106 with Alpha infections and 100 with B.1.1.214 infections (Table 3). 

**Table 3:** Characteristics of 206 households whose members were infected with SARS-CoV-2 B.1.1.214 or Alpha variants, Kyoto, Japan, November 2020–May 2021*

Variables	Alpha	Non-VOC	p value
No. households	106	100	
Median household size	3	4	
IQR	3–4	3–4	0.19
Range	2–10	2–9	
No. index cases	106	100	
Median age, y	38	47.5	
IQR	23–56	27.75–58	0.12
Range	5–93	7–91	
Sex, no. (%)			
M	73 (68.9)	70 (70.0)	0.95
F	33 (31.1)	30 (30.0)	
No. household contacts	282	282	
Median age, y	35.5	35.5	
IQR	13–53	16–52	0.96
Range	0–90	1–95	
Sex, no. (%)			
M	129 (45.7)	130 (46.1)	0.90
F	153 (54.3)	152 (53.9)	

*Alpha variant B.1.1.7 or non-VOC B.1.1.214. IQR, interquartile range; VOC, variant of concern.

Of the households with Alpha infections, we noted 106 index cases and 282 contacts; the median household size was 3 persons. Of 100 households with B.1.1.214 infections, we noted 100 index cases and 282 contacts, and the median household size was 4 persons. Contacts had RT-PCR testing a median of 6 (IQR 4–7) days after the index case was tested. Comparing lineages, we did not note statistically significant differences in the household size, age, or sex of the index cases and their contacts. We were able to determine lineages for 63 households with >2 (maximum of 5) members, and the lineages among each member were concordant in all households. The observed SAR in households with Alpha (62.4%) was higher than in households with B.1.1.214 (53.9%) (Table 4). In addition to differences between lineages, the observed SAR in each category was higher among adult (persons 19–59 years of age) index case-patients, elderly (persons >60 years of age) contacts, symptomatic index cases, and small household sizes (2–3 members) (Table 4). A risk factor analysis conducted by using a generalized linear mixed-effects model found that index case symptoms (adjusted odds ratio [aOR] 2.84, 95% CI 1.49–5.42; p<0.01) and Alpha lineage (aOR 1.52, 95% CI 1.06–2.18; p = 0.02) were significantly associated with household transmission (Table 4). Persons living in households of >4 members were at a lower risk for transmission. All model-predicted SARs were similar to the observed SARs, indicating the model was valid. 

**Table 4:** Secondary attack rates and risk factors for SARS-CoV-2 infection in 206 households whose members were infected with SARS-CoV-2 B.1.1.214 or Alpha variants, Kyoto, Japan, November 2020–May 2021*

Variables	No. contacts	No. infected	Secondary attack rate, %		Adjusted odds ratio (95% CI)†	p value†
			Observed	Predicted (IQR)†		
Overall	564	328	58.2	58.1 (46.5–71.1)	NA	NA
Index case age group, y						
<18	57	23	40.4	40.4 (28.3–49.8)	0.67 (0.35–1.28)	0.22
19–59	419	256	61.1	61.1 (51.7–70.9)	Referent	
>60	88	49	55.7	55.5 (44.9–64.0)	0.73 (0.44–1.22)	0.23
Contacts age group, y						
<18	167	82	49.1	49.1 (37.3–61.9)	0.72 (0.48–1.07)	0.10
19–59	344	208	60.5	60.4 (52.7–70.9)	Referent	
>60	53	38	71.7	71.9 (66.0–81.0)	1.75 (0.89–3.42)	0.10
Index case symptoms						
Asymptomatic	58	19	32.8	32.7 (24.1–43.4)	Referent	
Symptomatic	506	309	61.1	61.1 (51.7–70.9)	2.84 (1.49–5.42)	<0.01
Household size						
2–3	171	123	71.9	71.9 (70.9–78.7)	Referent	
4	189	112	59.3	59.3 (52.0–69.3)	0.61 (0.38–0.97)	0.04
5	100	43	43.0	42.9 (37.3–47.5)	0.34 (0.20–0.58)	<0.01
>6	104	50	48.1	48.0 (44.4–55.3)	0.46 (0.26–0.79)	<0.01
SARS-CoV-2 lineage						
Non-VOC, B.1.1.214	282	152	53.9	54.0 (44.9–64.0)	Referent	
Alpha, B.1.1.7	282	176	62.4	62.3 (55.3–72.7)	1.52 (1.06–2.18)	0.02

*IQR, interquartile range; NA, not applicable; VOC, variant of concern. †Calculated by using a generalized linear mixed-effects logistic regression model.

We performed a phylogenetic analysis of 135 Alpha and 143 B.1.1.214 genomes obtained from all 206 households (Appendix 2 Figure). Among these genomes, 127 were obtained from >2 household members. The average number of single-nucleotide polymorphism (SNP) differences among genomes from each household was smaller than SNP differences among other genomes in the corresponding lineage. B.1.1.214 genomes had a median of 0 (range 0–10) SNP differences among household members versus 14.3 SNPs from genomes outside the household, and B.1.1.7 genomes had a median of 0.7 (range 0–5) SNP differences within households versus 6.6 SNPs from genomes outside the household.

## Discussion

Kyoto and Japan experienced 4 waves of COVID-19 and the prevalent lineages in Kyoto’s 4 waves were similar to those in Japan (Figure 2). Before the first wave (January–March 2020), SARS-CoV-2 lineages A and B, which caused the initial outbreak in China, were introduced to Japan, then lineage B.1 or B.1.1 was introduced from Europe (*15*). The B.1 derivatives have a spike protein D614G mutation, resulting in increased transmissibility (*16*); this mutation subsequently resulted in worldwide spread and replacement of other existing lineages (*17*). The first wave in Japan was characterized by B.1/B.1.1 and its derivatives, which evolved and spread domestically (*15*). Among these lineages, B.1.48 was a domestic lineage that has not been reported outside Japan. The B.1.48 lineage was the second most common lineage in Kyoto and had a higher prevalence in that city than in Japan overall. This finding suggests a local outbreak, although the sample size during the first wave in Kyoto was limited. The second and third waves were caused by 2 domestic B.1 derivatives, B.1.1.284 in the second wave and B.1.1.214 in the third wave (*18*). These lineages did not harbor mutations in the spike protein, and explanations for lineage replacement are lacking. Between the third and fourth waves, during March 2021, the R.1 lineage, which was predominantly found in the United States, replaced B.1.1.214 in Kyoto and in Japan. R.1 harbors the spike protein mutation E484K, which is associated with immune escape and an increased reproduction number (*19*). The global origin of R.1 currently is unknown, but it was possibly imported from a country where the presumptive ancestor of B.1.1.316 was circulating (*18*). R.1 was replaced by the Alpha variant, which was responsible for the fourth COVID-19 wave in Japan. The Alpha variant was first detected in England and caused a global pandemic because of its higher transmissibility (*8*,*20*).

Genomic sequencing to detect variants has been performed worldwide at an unprecedented rate, but the coverage of samples is still biased toward regions and countries with high testing and sequencing capacity (*21*). In Japan, genome sequencing is performed under governmental leadership in national or regional infectious disease laboratories or large-scale private laboratories. By June 28, 2021, ≈7% of SARS-CoV-2–positive samples had been analyzed (*22*). We determined the genomes of 20% of cases in Kyoto through a collaboration between the local health department and a university hospital. In addition to genomic surveillance, the collaboration included mass PCR testing needed for epidemiologic investigations, mass screening for SARS-CoV-2 antibodies among essential workers, and establishing COVID-19 infection control programs targeting small-scale hospitals and facilities for elderly or disabled persons.

With the all-case investigation strategy, we were able to test 87.1% of case-contacts. By contrast, data from US public health authorities reported that 59% of US cases had been investigated, 71% of contacts were notified, and only 14.1%–54.7% of contacts had been tested (*23*). We found the RT-PCR test positivity rate of contacts in Kyoto was the highest among household members (24.9%). In addition, the positivity rate among family members living separately was 14.5%, which was similar to the average in the general population (15.1%), indicating that households were the main transmission venues during the third COVID-19 wave in Kyoto (Table 1). The importance of RT-PCR testing of close contacts was confirmed by the higher test positivity rate (15.1%) among close contacts than among the general population (5.9%).

The incidence of clusters was higher during the fourth wave than the third wave in Kyoto (Table 2). However, the frequency of cluster-associated cases and the number of cases per cluster was lower in the fourth wave. Clusters occurred in hospitals and LTCFs and were related to inappropriate use of personal protective equipment (*3*). Clusters also were reported from other congregate settings, such as house parties, homeless shelters, and food processing facilities (*24*). As described, we interceded in hospitals and adult daycare and LTCFs to improve infection prevention measures, which might have contributed to the decreased incidence of clusters and numbers of cases per cluster in those settings. 

Two doses of a COVID-19 vaccine (BNT162b2 [Pfizer-BioNTech, https://www.pfizer.com] or mRNA-1273 [Moderna, https://www.moderna.com]) are highly effective against the Alpha variant and non-VOCs (*25*). Japan designated healthcare workers as a vaccine priority group and began a vaccination program for them on May 2021; vaccination for residents of LTCFs began in March 2021. By the end of May 2021, 12.2% of Kyoto citizens had received >1 dose of a vaccine, and 3.2% had received 2 doses. These vaccination data suggest that vaccination might be associated with the reduction in cluster-associated cases but might not be associated with the number of overall cases during the fourth wave.

Household secondary cases accounted for 26.7%–28.9% of all COVID-19 cases in Kyoto, and these rates increased during the fourth wave compared with the third wave. Similarly, a study conducted in Canada at the beginning of the pandemic (January–July 2020) reported a 20.5% rate of household secondary cases (*5*). During the third and fourth COVID-19 waves in Kyoto, the city declared a state of emergency and residents were advised to stay at home. This stay-at-home recommendation might have contributed to the suppression of community spread but also might be associated with the increased rates of household transmission. A modeling study in China estimated that 51.5% of infections occurred in households during the first outbreak, and this number increased to 69.8% after quarantine (*7*). During lockdown in the United States, household transmission was estimated to increase 25%–50% (*26*).

The SARs we found in Kyoto were higher than other reported SARs, probably reflecting differences in lineages, transmission opportunities, and case investigation strategies. We assumed a very low possibility of transmission from outside the household during quarantine because public health centers issued a strong request for citizens to stay home. Data from 10 prefectures of Japan, not including Kyoto, reported a 19.0% SAR during the first wave, in which imported and cluster-associated cases were the main sources of transmission, but we noted a 53.9% SAR for B.1.1.214 in this study (Table 4). Those data were mostly generated from cases during the third wave, during which household transmission increased because of the state of emergency and prevalence of different lineages with potentially superior transmissibility. A meta-analysis of worldwide data estimated that SARs increased over time from 13.4% during January–February 2020 to 31.1% during July 2020–March 2021 and that these increases might be associated with the spread of variants with increased transmissibility (*27*). That report also noted SARs as high as 24.5% (range 10.9%–46.2%) for Alpha variants (*27*). In our cohort, household infection with Alpha clearly was associated with an increased SAR of 62.4% and a higher risk for transmission with an aOR of 1.52 (Table 4), which probably contributed to the larger number of household secondary cases during the fourth wave (Table 2). Delta variants are associated with a higher risk for household transmission than Alpha variants and have an aOR of 1.70 (*28*), which implies a further increase in SAR and effects of household transmission. Vaccination lowers the risk for household transmission of VOCs and can be a vital strategy for reducing infections (*28*,*29*). However, the Delta and Omicron VOCs that emerged after Alpha have the potential for immune escape; observational studies suggest that vaccine effectiveness against Delta was lower than for Alpha (*30*) and that effectiveness against Omicron is further lower than that for Delta (*31*). In addition, postvaccine immunity could wane over time (*25*,*31*). Proposed booster doses could improve vaccine effectiveness, even against Delta and Omicron variants (*25*,*32*). Improved vaccination programs that include booster doses and evaluation studies of vaccine effectiveness in households could help reduce household transmissions.

In addition to VOCs, the risk factors for household transmission include age, fewer household members, contact frequency, and symptomatic index cases (*5*,*14*,*33*–*35*). Our results are consistent with previous reports that show a lower risk for persons <18 years of age to be index cases and higher risk for transmission among adult contacts, small households, and symptomatic index cases (*5*,*14*,*33*–*35*) (Table 4). In an outbreak in China, interventions targeting households, mass isolation of patients, quarantine of household contacts, and movement restriction policies succeeded in reducing the reproduction number of index cases by 52% and secondary cases by 63% (*35*). Public health interventions targeting households, such as public health messaging, self-quarantine at home (*35*), and promoting isolation facility use (*5*), appear to be effective strategies for reducing the number of COVID-19 cases.

The strengths of this study include a high coverage of epidemiologic investigations, which were supported by a high proportion of testing among close contacts and cluster-associated contacts, as well as genomic surveillance. The first limitation of the study is that differences among the study periods in the analyses of the cases and contacts (Tables 1–4) should be noted when interpreting and generalizing the results in combination. Second, we could not perform risk factor analyses for RT-PCR test positivity among close contacts and those of the cluster-associated cases because of the absence of detailed epidemiologic data, including clinical symptoms, vaccination status, infection prevention measures, and RT-PCR testing delays. Thus, the effects of different variants on close contact categories other than households and on cluster-associated cases were not elucidated. 

In conclusion, this study elucidates the epidemiologic characteristics of COVID-19 patients and their contacts in Kyoto, Japan, and highlights the role of household transmission, as enhanced by the Alpha variant, by using viral genomic analysis. In addition to current epidemiologic investigation efforts, including contact tracing, strengthening interventions that target household are needed for infection control. Continued collaboration between public health departments and academia can accurately illuminate the epidemiology of COVID-19 and whether emerging VOCs have higher transmissibility.

Appendix 1 Accession numbers for SARS-CoV-2 genomes used in a study of transmissibility of SARS-CoV-2 B.1.1.214 and Alpha variants during 4 COVID-19 waves, Kyoto, Japan, January 2020–June 2021.

Appendix 2 Additional information on transmissibility of SARS-CoV-2 B.1.1.214 and Alpha variants during 4 COVID-19 waves, Kyoto, Japan, January 2020–June 2021.
