# Supplementary material for: Transmissibility of SARS-CoV-2 B.1.1.214 and Alpha Variants during 4 COVID-19 Waves, Kyoto, Japan, January 2020–June 2021
Source: Emerg Infect Dis. 2022 Aug;28(8):1569–77. doi: 10.3201/eid2808.220420 (PMC9328921; doi:10.3201/eid2808.220420)
Supplement: Appendix 2 — Additional information on transmissibility of SARS-CoV-2 B.1.1.214 and Alpha variants during 4 COVID-19 waves, Kyoto, Japan, January 2020–June 2021. [file 22-0420-Techapp-s2.pdf]

Article DOI: https://doi.org/10.3201/eid2808.220420

Transmissibility of SARS-CoV-2 B.1.1.214 and Alpha Variants during 4 COVID-19 Waves, Kyoto, Japan, January 2020–June 2021

Appendix 2

Appendix 2 Table. Settings of outbreak clusters during the third and fourth COVID-19 waves, Kyoto, Japan

Setting	Third wave	Fourth wave
Aged care facility*	27	7
Hospital	11	6
Office	0	13
School	2	9
Preschool or nursery facility	4	4
Dining together	5	2
Sports team	0	2
Karaoke	0	1

*Includes long-term care and day care facilities for adults.

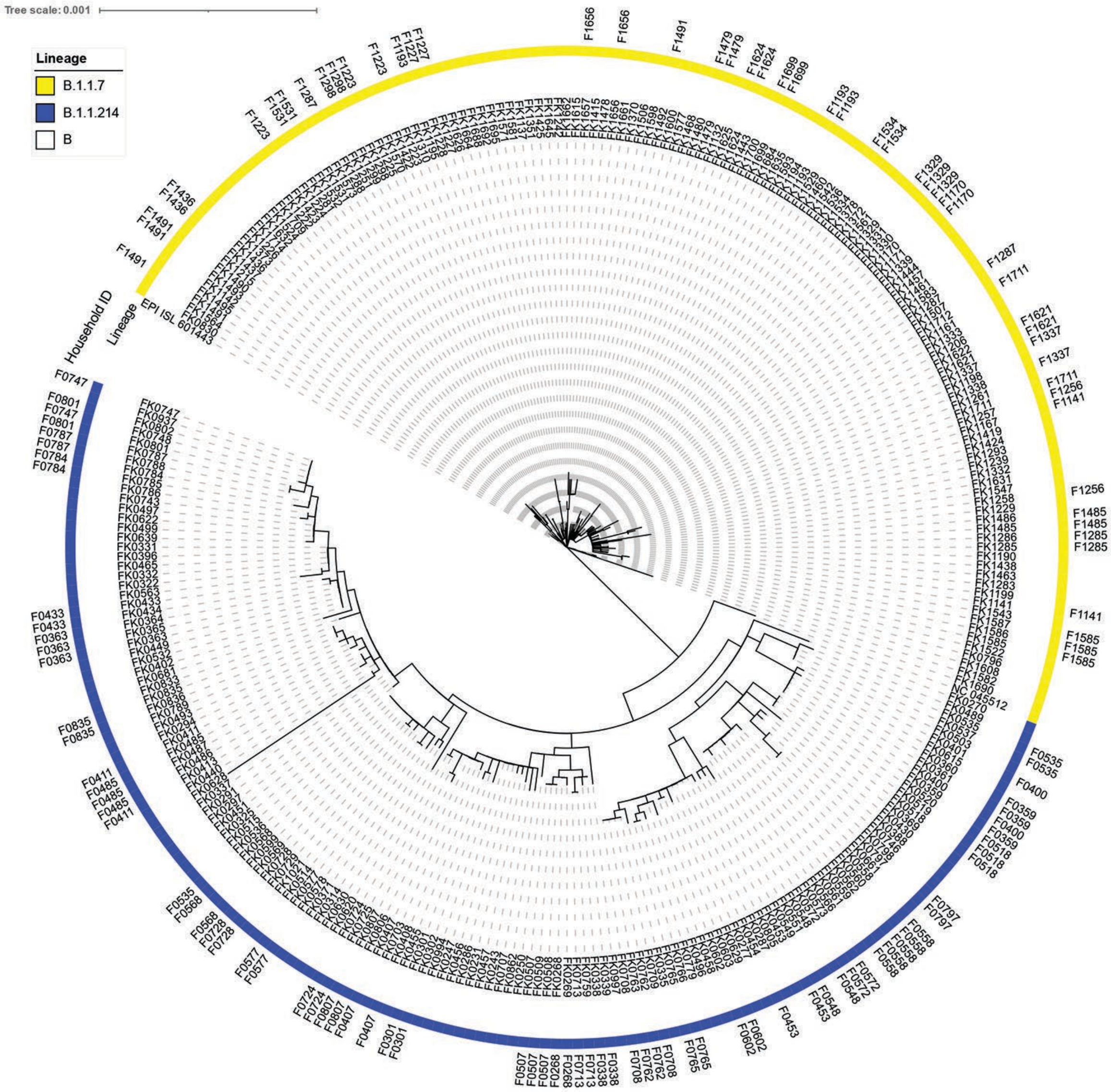

Appendix 2 Figure. Maximum-likelihood phylogenetic tree of SARS-CoV-2 strains obtained from 278 COVID-19 cases from 206 households in Kyoto, Japan, November 2020–May 2021. The tree was constructed by using the best-fit nucleotide substitution of the general time-reversible plus gamma distribution plus invariable site model determined by ModelFinder (IQ-TREE, http://www.iqtree.org). Two reference strains for lineages B (GenBank accession no. NC_045512) and B.1.1.7 (GISAID accession no. EPI_ISL_601443) are also included. Household identifiers are noted around the outermost layer of the tree.
